# Family caregivers’ abusive behaviour and its association with internalized stigma of people living with schizophrenia in China

**DOI:** 10.1038/s41537-023-00393-6

**Published:** 2023-09-19

**Authors:** Yilu Li, Dan Qiu, Qiuyan Wu, Anyan Ni, Zixuan Tang, Shuiyuan Xiao

**Affiliations:** 1 Department of Social Medicine and Health Management, Xiangya School of Public Health, Central South University, Changsha, Hunan China; 2 National Clinical Research Center for Mental Disorders and Department of Psychiatry, The Second Xiangya Hospital of Central South University, Changsha, Hunan China

**Keywords:** Schizophrenia, Human behaviour

## Abstract

Family caregiving of people living with schizophrenia (PLS) can be burdensome, and some family caregivers may perpetrate abusive behavior that could be harmful to PLS. This study aims to examine the association of family caregivers’ abusive behavior with internalized stigma of PLS and draw attention to this problem. PLS were recruited from four cities across China and completed measures of abusive behavior and internalized stigma. Linear regression analyses were used to determine the association between family caregivers’ abusive behavior and internalized stigma of PLS. A total of 693 PLS were include in this study. 22.7% of the participants had experienced one or more of the abusive behaviors perpetrated by family caregivers. The most common type of abusive behavior towards PLS was verbal abuse and 4.2% of the participants reported physical abuse. 44.6 % of participants reported a high level of internalized stigma. PLS who experienced any abusive behavior by family caregivers had significantly higher levels of internalized stigma. Family caregivers’ abusive behavior is positively associated with alienation and social withdrawal but not with stereotype endorsement and discrimination of PLS. To end all forms of stigma and discrimination against PLS, more attention needs to be paid to the families of PLS.

## Introduction

There was a persistent public stereotype that people living with schizophrenia (PLS) were violent and dangerous. PLS were often identified as typical perpetrators of violence. In fact, PLS were more likely to experience violence in their lives than to perpetrate it^1–3^. A systematic review of the prevalence of victimization in people with a psychotic disorder suggested that two-thirds of PLS were found to have been a victim of violent victimization during their entire adulthood, which was 4–6 times higher than in the general population^4^. Family abuse accounted for a greater proportion of the total violent victimization experienced by PLS^2,5–7^. However, far more studies have focused on violence perpetrated by PLS against family members than vice versa.

Family abuse has been recognized as a major public health problem worldwide. In the child and elderly populations, abuse by family caregivers has been a public health priority because of its significant negative consequences, including injury, chronic physical illness, increased rates of depression, anxiety, and suicidal behavior of care recipients^8,9^. Family abuse can be manifested in many ways and can be categorized into 5 types: physical, verbal or psychological, sexual, financial, and neglect^10,11^. Abuse that occurs in a family setting may be particularly related to the caregiver-recipient relationship, family functioning, and family caregiving burden^10,12^. Previous studies has indicated that care recipients with psychological or behavioral symptoms may exacerbate caregiver-recipient conflicts and result in family abuse^12^. In addition, abuse may also occur when the care recipients are highly dependent on the family and the burden of caregiving is excessive^12,13^. Thus, PLS may also be victims of family abuse^14^. A study based on Crime Survey for England and Wales estimated that 30% to 60% of people with severe mental illness reported violence perpetrated by families during their lifetime^15^. A scoping review found that the “perpetrators” of microaggressions (brief and commonplace daily verbal, behavioral, or environmental indignities that communicate hostile, derogatory, or insults) toward people with mental illness were predominantly family members, rather than strangers or acquaintances^16^. Researchers have described how PLS experienced abusive behavior from their family caregivers in some qualitative studies^14,17,18^. Those studies explored the nature, types, and experiences of abusive behavior by family caregivers toward PLS, but rarely reported its prevalence and studied its consequences.

In order to bring more attention to this problem, understanding the consequences of family caregivers’ abusive behavior is important. A large body of research indicates that individuals exposed to family abuse experience psychological, social, physical, and cognitive consequences^19^. An important consequence of family abuse of PLS that has been ignored in the existing literature may be internalized stigma. Internalized stigma occurs when PLS are aware of the negative stereotypes of schizophrenia, agree with the stereotypic or stigmatizing views, and turn them against themselves^20^. According to a situational model of stigma, if PLS make sense of the negative reactions of others and perceive the negative reaction as legitimate, they will internalize stigma and have diminished self-esteem^21^. The negative reactions and unfair treatment from others were significant in the process of internalization of stigma. For PLS, they are most likely to experience negative reactions and unfair treatment in the family, because family caregivers are actually on the front line of care provision^22,23^. A number of PLS reported negative reactions and unfair treatment from family, including being “scolded or yelled at” and being hit by family caregivers because of their illness^17,24^. Those PLS who have been scolded or hit by family caregivers may be aware of their stigmatized condition, feel inferior to others, lose self-esteem, or feel shame^14^. Violence victimization of PLS is also significantly associated with social withdrawal^25^. However, most of the research explores sociodemographic and illness-related factors of internalized stigma rather than considering the influence of family^26^. The fragmentation of efforts to address the causes of internalized stigma and understand the family experiences of PLS has hindered progress to date.

To explore abusive behaviors by family caregivers does not mean to blame them. Rather, this study seeks to understand conflict within the family of PLS in order to improve anti-stigma interventions and family caregiving in the future. By carrying out an analysis of cross-sectional data from a community-based study in China, this study aimed at (1) describe the pattern of family caregivers’ abusive behavior against PLS in China (including psychological and physical abuse); (2) investigate the association between family caregivers’ abusive behavior and internalized stigma of PLS.

## Method

### Participants and procedure

Considering the quality of community mental health services and economic status, 4 cities (Changsha, Wuhan, Guangzhou, Shenzhen) across southern China were selected as survey sites. This study was a multi-site, cross-sectional survey utilizing cluster sampling. First, two districts (one central and one suburban) were selected from each city. All affiliated community health service centers in the selected districts were included in the sampling frame (a total of 122 centers). Second, 50% of the community health service centers from each district were randomly selected and invited to participate in this study. Finally, a total of 45 community health service centers with agreement were included as survey sites. People with schizophrenia were registered in the “China Basic Public Health Services” program, which provided mental healthcare in community health service centers. Within each community health service center, healthcare workers were asked to identify all service users with a clinical diagnosis of schizophrenia. Inclusion criteria included: (1) participants were diagnosed as having schizophrenia by certified psychiatrists according to ICD-10 criteria; (2) aged 18 or older; (3) be currently living with their family members in the community (4) able to understand, read, and communicate with the investigators in Chinese. The sample size was calculated according to the form for cross-sectional study: *n* = Z^2^ P (1-P)/E^2^, where P (the prevalence of family abusive behavior) was estimated at 60% based on past studies^15^, Z was set as 1.96 at a confidence interval of 95%, an allowable error was set as 5%, the final sample size came to 369.

Data were collected by trained researchers from May 2021 to December 2021. Eligible participants were invited to complete the face-to-face interviews in the community health center. For participants who agreed to participate in the study but were reluctant to visit the community health center, researchers went door-to-door to participant homes companies by a community health worker. Both the clinical assessment and survey were implemented by the research team, including three students and a mental health professional who worked in the community. The research team received a 2-week clinical assessment training before the formal investigation and tested with high inter-rater reliability. Participants were offered CNY 20 ($3) as transportation reimbursement for taking part in the interview, which covered transportation costs for PLS and their families to travel to community health centers.

Out of the 972 PLS approached across 45 community health service centers, 104 PLS lived without any family members. Among 868 PLS who lived with family members in the community, 175 respondents have missing data on socio-demographic characteristics and internalized stigma. Compared with analyzed respondents, the respondents with missing data had non-significant differences in gender, age, illness duration, education level, marital status, and severity of symptoms. Rural respondents and respondents with poor functioning and disability had relatively more missing data on internalized stigma (see details in Supplementary Table 1). Finally, 693 participants with no missing data were included in the following analysis.

### Measurements

#### Abusive behaviors in caregiving

In this study, abusive behaviors are measured by the Modified version of the Conflict Tactics Scale (MCTS)^27^, including five indicators of psychological abuse (screamed or shouted, used a harsh tone of voice, insulted, swore or called them names; threatened to send them to hospital/nursing home, to stop taking care of or abandon, threatened to use physical force) and five indicators of physical abuse (withholding food, hitting or slapping, shaking, handling roughly in other ways, feeling afraid that the caregiver might hit or try to hurt the care recipient). This scale assesses how often in the past three months the caregivers had acted in each abusive behaviors towards the care recipient. Items are scored on a 4-point Likert scale from 0 (never) to 4 (all the time). A score of ≥1 for any question is classified as experienced abusive behaviors. MCTS had good sensitivity and specificity when used as a screening instrument for abuse, and be widely used in research on carer abusive behaviors^27–29^. MCTS have comparable psychometric performance in terms of the internal consistency, convergent validity, and known group’s validity in the sample of this study. The Cronbach’s Alpha of MCTS for the sample was 0.913. Convergent validity was in general accordance with expectations. MCTS had significant positive correlations with severity of symptoms (*r* = 0.15, *p* < 0.01) and family interactions score (*r* = 0.13, *p* < 0.01), and significant negative correlations with quality of life among PLS (*r* = −0.16, *p* < 0.01), respectively. For known groups’ validity, PLS who had aggressive behavior in the past 2 months reported higher MCTS scores than those without aggressive behavior, which is consistent with previous studies^1,28^.

#### Internalized stigma

The Internalized Stigma of PLS was assessed by The Internalized Stigma of Mental Illness Scale (ISMI), a 29-item self-report scale. ISMI is designed to measure the subjective experience of stigma, with subscales measuring Alienation, Stereotype Endorsement, Perceived Discrimination, Social Withdrawal, and Stigma Resistance. Item is rated on a 4-point Likert scale from 1 (strongly disagree) to 4 (strongly agree). Higher total scores of ISMI are indicative of higher levels of internalized stigma. Several research has indicated that the ‘stigma resistance’ subscale is a separate construct that different to the other subscales^30^. Therefore, in this study, internalized stigma refers to the summed average of the other four ISMI subscales. A cut-off points at 2.5 and above on the mean item score of ISMI was utilized to categories high level of internalized stigma^31,32^. This cut-off points of 2.5 have been used in several other research^32–34^. The Chinese version of ISMI showed great reliability and validity for assessment of internalized stigma^35^.

#### Socio-demographic and illness-related characteristics

Socio-demographic characteristics and illness-related characteristics of PLS were collected by face-to-face interviews. Sociodemographic characteristics of PLS including sex, age, living area(urban/rural), marital status (single, married/cohabiting, separated/divorced/widowed), education (primary or below, secondary, college/university), employment (unemployed, full-time, part-time, retired) and illness duration. The urban-rural classification of PLS residence in this study is from the household registration system, which is officially designated by the Chinese government. Besides, respondents reported their household monthly income per capita. Household poverty was defined as monthly per capita household income below the poverty threshold. Given the huge income disparity in China, the poverty standard in this study was the minimum living standard defined by the local government in 2021. The minimum living standard in each city was: 1300RMB/month for Shenzhen, 1080RMB/month for Guangzhou, 750 RMB/month for Changsha, and 870RMB for Wuhan.

The 12- item World Health Organization Disability Assessment Schedule second version (WHODAS 2.0) was used to assess functioning and disability in major life domains of PLS^36^. Items can be scored on a 5-point scale ranging from 1 = none to 5 = extreme/cannot do. The higher scores reflect greater disability. The Chinese version of WHODAS 2.0 was found to have high internal consistency and test-retest reliability^37^.

The Brief Psychiatric Rating Scale (BPRS) was used to assess the severity of psychotic and depressive symptoms in psychotic disorders [25]. The 18-item version of the scale was used in this study, which assesses symptoms including somatic concern, anxiety, emotional withdrawal, conceptual disorganization, guilt feelings, tension, mannerisms and posturing, grandiosity, depressive mood, hostility, suspiciousness, hallucinatory behavior, motor retardation, uncooperativeness, unusual thought content, blunted affect, excitement, and disorientation. Each item is rated on an 8-point scale ranging from 0 = not present and 7 = extremely severe. The BPRS total scores range from 0 to 126, with a higher total score representing more severe psychotic symptoms.

### Statistical analysis

All analyses were performed using SPSS version 23 (IBM Corporation, Armonk, NY, USA). Continuous variables were described using mean and standard deviation, categorical variables are presented as count and percent. The t-test and analyses of variance (ANOVA) examined the differences in mean scores of ISMI. Categorical variables were compared using the linear by-linear association chi-test. Three linear regression analyses were used to determine the association between family caregivers’ abusive behavior and overall scores of ISMI. Some previous studies indicated that female PLS^5^, PLS with younger age^4^, PLS being single^4^, PLS with higher level of disability and severity of symptoms^38^, PLS with poor financial situation and unemployment^1^ reported more violence victimization. Thus, gender, age, residence (urban or rural), education, marriage status, employment, household poverty, illness duration, disability, and severity of symptoms of PLS were selected as covariates. To avoid multicollinearity, we checked the variance inflation factors (VIF) for variables and excluded the variables with VIF greater than 10 from the model. In model 1, only the gender and age of PLS were adjusted. In model 2, additional factors of residence (urban or rural), education, marriage status, employment, household poverty, and illness duration were included. Model 3 adjusted for model 2 plus disability and severity of symptoms in PLS. The association between family caregivers’ abusive behavior and each component of ISMI was evaluated using linear regression analyses. Additionally, the associations between abusive behavior and the level of internalized stigma were investigated in the multivariable logistic regression model, and the effect sizes were shown with odds ratios (ORs) and their 95% confidence intervals (CIs). Statistical significance was set at 0.05.

## Result

### Participant characteristics

The sociodemographic characteristics of the participants are shown in Table 1. Overall, 693 PLS living with family caregivers were included in the analysis and 55.4% of participants were female. The majority of participants live in urban areas (86.0%). The mean age of participants was 46 (S.D. = 12.9) years old, with 74.8% of participants have lived with schizophrenia for more than 10 years. The vast majority of the participants had a secondary level of education (61.9%) and were unemployed (71.1%). More than half of the participants (52.5%) were married or cohabiting and 21.9% of the participants were living in household poverty. The BPRS had a mean score of 22.02 (S.D. = 2.15) and WHODAS had a mean score of 24.00 (S.D. = 12.18).

**Table 1:** Socio demographic characteristics, stigma, and abusive behaviour of participants.

	Total N (%)	High Internalized Stigma			Experienced abusive behavior		
		*N* (%) or M(SD)	chi	*p*	*N* (%) or M(SD)	chi	*p*
Residence			10.554	0.001		0.896	0.457
Urban	596 (86.0)	251 (42.1)			136 (22.8)		
Rural	97 (14.0)	58 (59.8)			21 (21.6)		
Gender			0.245	0.645		0.648	0.324
Male	309 (44.6)	141 (45.6)			67 (21.7)		
Female	384 (55.4)	168 (43.8)			90 (23.4)		
Age, years			3.659	0.454		5.443	0.245
18–30	83 (12.0)	34 (41.0)			25 (30.1)		
31–40	182 (26.3)	88 (48.4)			38 (20.9)		
41–50	162 (23.4)	75 (46.3)			41 (25.3)		
51–60	179 (25.8)	71 (39.7)			33 (18.4)		
>60	87 (12.6)	41 (47.1)			20 (23.0)		
Illness duration, years			7.947	0.094		3.097	0.542
1–5	80 (11.5)	26 (32.5)			21 (26.3)		
6–10	95 (13.7)	37 (38.9)			16 (16.8)		
11–20	252 (36.4)	122 (48.4)			60 (23.8)		
21–30	161 (23.2)	76 (47.2)			34 (21.1)		
>30	105 (15.2)	48 (45.7)			26 (24.8)		
Education			0.514	0.773		0.485	0.785
Primary or below	188 (27.1)	84 (44.7)			42 (22.3)		
Secondary	429 (61.9)	194 (45.2)			100 (23.3)		
College/university	76 (11.0)	31 (40.8)			15 (19.7)		
Marital Status			2.881	0.237		2.128	0.345
Single	258 (37.2)	109 (42.2)			65 (25.2)		
Married or cohabiting	362 (52.2)	161 (44.5)			74 (20.4)		
Separated /Divorced /Widowed	73 (10.5)	39 (53.4)			18 (34.7)		
Employment			15.305	0.002		3.025	0.388
Unemployed	494 (71.3)	235 (47.6)			117 (23.7)		
Retired	71 (10.2)	30 (42.3)			17 (23.9)		
Part-time	23 (3.3)	14 (60.9)			6 (26.1)		
Full-time	105 (15.2)	30 (28.6)			17 (16.2)		
Household poverty			0.169	0.712		0.099	0.827
no	541 (78.1)	239 (44.2)			124 (22.9)		
yes	152 (21.9)	70 (46.1)			33 (21.7)		
BPRS	22.02 (7.15)	23.90 (8.74)	−6.381	<0.001	23.63 (9.26)	−3.235	0.001
WHODAS 2.0	24.00 (12.18)	28.34 (12.55)	−8.880	<0.001	23.68 (12.19)	0.378	0.705
Experienced abusive behavior			8.561	0.004			
no	536 (77.3)	256 (42.5)			/		
yes	157 (22.7)	53 (58.9)			/		
Internalized stigma						8.528	0.005
low internalized stigma	384 (55.4)	/			71 (18.5)		
high internalized stigma	309 (44.6)	/			86 (27.8)		

### Abusive behavior by family caregivers

Overall, 157 (22.7%) of the participants had experienced one or more of the abusive behaviors perpetrated by family caregivers. Specifically, 21.7% of male participants and 23.4% of female participants had experienced abusive behavior. The most common types of abusive behaviors included yelling and screaming at the PLS (21.5%) and using a harsh voice/ insulting/ calling the PLS names/ swearing at the PLS (15.9%). In addition, 4.2% of participants had been hit/slapped/shook by family caregivers. The details of abusive behaviors by family caregivers are shown in Table 2.

**Table 2:** Abusive Behavior by Family Caregivers towards Care Recipient.

Abusive Behavior	Never	Almost never	Sometimes	Most of time	Always
Screamed and yelled at	544 (78.5)	66(9.5)	53 (7.6)	26 (3.8)	4 (0.6)
Used harsh tone of voice, insulted, called names, swore at	583 (84.1)	41 (5.9)	42(6.1)	23 (3.3)	4 (0.6)
Threatened to send to hospital/nursing home	649 (93.7)	15 (2.2)	17 (2.5)	9 (1.3)	3 (0.4)
Threatened to stop taking care of or abandon	652 (94.1)	16 (2.3)	17 (2.5)	6 (0.9)	2 (0.3)
Threatened to use physical force	659 (95.1)	11 (1.6)	14 (2.0)	8 (1.2)	1 (0.1)
Afraid caregiver might hit or try to hurt	654 (94.4)	9 (1.3)	17 (2.5)	10 (1.4)	3 (0.4)
Withheld food	679 (98.0)	2 (0.3)	7 (1.0)	4 (0.6)	1 (0.1)
Hit or slapped	665 (96.0)	9 (1.3)	12 (1.7)	6 (0.9)	1 (0.1)
Shook	664 (95.8)	10 (1.4)	12 (1.7)	6 (0.9)	1 (0.1)
Handled roughly in other ways	665 (96.0)	13 (1.9)	7 (1.0)	7 (1.0)	1 (0.1)

Table 1 shows the distribution of the experienced abusive behavior of PLS. The experienced abusive behaviors have non-significant differences between each socio-demographic characteristics group of participants. PLS who have experienced abusive behavior by family caregivers reported a higher mean scores of the internalized stigma.

### Internalized stigma

Table 3 presents the overall scores of ISMI and the scores of each subscale. The mean score of overall internalized stigma was 2.48 (S.D. = 0.71), with 44.6 % of participants reporting a high level of internalized stigma. For each component of internalized stigma, the highest mean scores for social withdrawal 2.60 (S.D. = 0.88), followed by the alienation 2.53 (S.D. = 0.88), discrimination experience 2.48 (S.D. = 0.84) and stereotype endorsement 2.33 (S.D. = 0.72).

**Table 3:** Internalized stigma total and subscales score in PLS.

	Total	PLS with AB	PLS without AB	*t*	*p*
Total of ISMI	2.48 (0.71)	2.60 (0.62)	2.44 (0.73)	−2.514	0.012
Alienation	2.53 (0.88)	2.69 (0.79)	2.48 (0.90)	−2.576	0.010
Stereotype endorsement	2.33 (0.72)	2.40 (0.64)	2.31 (0.74)	−1.430	0.153
Discrimination experience	2.48 (0.84)	2.58 (0.79)	2.45 (0.85)	−1.787	0.074
Social withdrawal	2.60 (0.88)	2.77 (0.80)	2.55 (0.89)	−2.727	0.007

*ISMI* The Internalized Stigma of Mental Illness scale, *AB* abusive behavior.

The overall level of internalized stigma of PLS who have experienced abusive behavior was significantly higher than PLS without any abusive behavior experience (2.60 V. S 2.44, *p* = 0.012). For each subscale, PLS with experienced abusive behavior reported higher scores on alienation (2.69 V. S 2.48, *p* = 0.010) and social withdrawal (2.77 V. S 2.55, *p* = 0.007), but not on stereotype endorsement (2.40 V. S 2.31, *p* = 0.153) and discrimination (2.58 V. S 2.45, *p* = 0.074).

### Association between abusive behavior and internalized stigma

The results of multivariate linear regression on abusive behavior and internalized stigma are summarized in Table 4. In the full-adjusted model, family caregivers’ abusive behavior is significantly associated with internalized stigma of PLS (b = 0.156, 95CI: 0.044 to 0.268). Participants who experienced any abusive behavior by family caregivers had significantly higher levels of internalized stigma. The results of logistic regression analysis show that PLS with family abusive behaviors had 1.77 (95% CI: 1.20–2.36) times greater likelihood of a high level of internalized stigma (see details in Supplementary Table 2).

**Table 4:** Multiple regression analyses of internalized stigma and caregivers’ abusive behavior.

	Model 1		Model 2		Model 3	
	B (95%CI)	*p*	B (95%CI)	*p*	B (95%CI)	*p*
Total of ISMI	0.166 (0.040 to 0.292)	0.010	0.145 (0.022 to 0.268)	0.021	0.156 (0.044 to 0.268)	0.006
Alienation	0.210 (0.053 to 0.366)	0.009	0.187 (0.031 to 0.342)	0.019	0.200 (0.054 to 0.347)	0.008
Stereotype endorsement	0.097 (−0.031 to 0.225)	0.136	0.071 (−0.054 to 0.195)	0.268	0.081 (−0.034 to 0.197)	0.168
Discrimination	0.145 (−0.005 to 0.294)	0.058	0.124 (−0.023 to 0.271)	0.099	0.122 (−0.019 to 0.263)	0.089
Social withdrawal	0.221 (0.065 to 0.376)	0.006	0.208 (0.053 to 0.362)	0.009	0.228 (0.084 to 0.371)	0.002

*CI* confidence interval.

Model 1 adjusted for age and gender.

Model 2 adjusted for model 1 plus residence, education, marriage status, employment, household poverty, and illness duration.

Model 3 adjusted for model 2 plus disability and severity of symptoms in PLS.

For each component of internalized stigma, family caregivers’ abusive behavior is positively associated with alienation (b = 0.200, 95CI: 0.054 to 0.347) and social withdrawal (b = 0.228, 95CI: 0.084 to 0.371). Family caregivers’ abusive behavior has non-significant association with discrimination experience and stereotype endorsement.

## Discussion

To our knowledge, no past studies have assessed abusive behaviors perpetrated by family caregivers toward PLS in China. This study indicated that 22.7% of PLS in China had experienced one or more of the abusive behaviors perpetrated by family caregivers. The most common types of abusive behaviors included yelling and screaming at PLS and using harsh voices/insults/curses at PLS. A study in rural China reported that 18.9% of PLS experienced at least one type of violent event, but did not specifically assess family violence^38^. While previous studies have not assessed abusive behaviors by family caregivers, a few qualitative studies have described some abusive behaviors experienced by PLS^17,18,39^. For example, a husband reported that he scolded and hit his wife when she had relapsed or was reluctant to do housework^17^. An older brother always scolds his ill brother for not going to work, which is seen as lazy and irresponsible^18^. Family abusive behaviors towards PLS are the most visible familial stigma of mental illness, but are rarely reported in the literature, especially in low- and middle-income countries^14^. Family abusive behaviors towards PLS are destructive and hidden. It is a taboo and difficult for both family members and PLS to acknowledge and report. As a result, the problem has been neglected for a long period of time. The manifestation, causes and consequences of family abusive behaviors toward PLS are rarely explored in the existing literature.

Abusive behaviors by family members toward persons with dementia have been an intensively studied topic in abuse research. Caregiving for PLS has many similarities with caregiving for dementia, such as high dependency on family caregivers to provide care^23,40^, inadequate support from the health care system^41^, heavy caregiver burdens^23,42^, stigma, and social exclusion^41,43^. Family caregiving for PLS shares these characteristics that may lead to abuse^12,44^, but few studies pay as much attention to family abuse of PLS as they do to abuse of persons with dementia. To raise public awareness of family abusive behaviors towards PLS, understanding its consequences is a priority. Therefore, this study attempts to understand the association between family caregivers’ abusive behaviors and PLS’ internalized stigma. The results of the analysis indicated that family caregivers’ abusive behaviors were significantly associated with PLS’ internalized stigma.

After controlling for several socio-demographic and illness-related characteristics of the participants, this study suggested that family caregivers’ abusive behaviors were associated with internalized stigma of PLS. PLS with family abusive behaviors had 1.77 times greater likelihood of a high level of internalize stigma. According to the What Matters Most theory, PLS may be initiated into a stigmatized role when traumatic interactions occur between PLS and family members^45^. Abusive behavior is a typical traumatic interaction within PLS families. When experiencing verbal or physical violence from family members, PLS may recognize and agree with their own stigmatized identity, develop thoughts of being inferior to others, reduce their self-esteem, and consequently avoid social contact. However, an earlier study in New York suggested that victimization of people with serious mental illness did not lead to perceived stigma^46^. The New York study recruited PLS from outpatient settings, did not describe the perpetrators of violence, and assessed only perceived stigma. Thus, the heterogeneity of the studies may contribute to different study settings and different measures of violence and stigma.

Because internalized stigma is a complex, culturally determined process with multiple components, the abusive behavior of family caregivers may have different effects on each component. Examining the differential associations between abusive behaviors and internalized stigma components may lead to a deeper understanding of the role they play in the process of stigmatization. Specifically, this current study found that family caregivers’ abusive behavior was positively associated with alienation and social withdrawal but not with stereotype endorsement and discrimination. There is considerable evidence that victims of family abusive behavior report higher levels of alienation and experience feelings of inferiority, uselessness, and disappointment^12,47,48^. The association between violence victimization and social withdrawal has also been found in previous studies^25,45,46^. It is worth noting that the abusive behavior of family caregivers is not significantly associated with stereotype endorsement and discrimination. In the study of the lived experience of PLS, many caregivers and PLS view abusive behaviors as a way of coping^16,24,41^. Under these circumstances and perceptions, PLS may not recognize that some relatively light abusive behaviors, such as yelling, constitute unfair treatment and discrimination. Besides, family caregivers’ abusive behavior may only reinforce one aspect of stereotype endorsement, such as beliefs about uselessness and inferiority, but not a stereotype such as PLS being violent. There is little research that explains how environmental factors, such as the family, influence the complex mechanisms of stigma. Little is known about the causes of stigmatization and stigma mechanisms. Gaps in mental health stigma research hinder the ability to understand the relationship between family abusive behaviors and internalized stigma. More qualitative research is needed to explore the subtleties and complexities nature of stigma and family caregivers’ abusive behavior that cannot be captured through direct questions.

There are several strengths in the present research. This study fills a gap in the literature about family caregivers’ abusive behavior and internalized stigma of PLS in China. This study was conducted in a community setting across four cities and has a relatively large sample size. However, several limitations should be taken into account. First, the study adopts cross-sectional study design, so it is not possible to examine the causality of the relationship between family caregivers’ abusive behavior and the internalized stigma of PLS. Second, self-report bias is always an issue when using interview methods to collect data and studying a problem such as abusive behavior and internalized stigma. It is hard to avoid social desirability when PLS self-reported their abuse experiences and stigma. Third, the generalizability of the findings is limited by the recruitment process. Some community health centers refused to participate in this study, and of 972 PLS approached in the study sites, only 693 completed the survey. Poorly functioning respondents may be excluded from the analysis because of their inability to understand the content of the ISMI and complete the assessment. Thus, the extent of internalized stigma and family caregivers’ abusive behavior may be underestimated. Additionally, this study only focuses on psychological and physical abuse in PLS family. Other types of abusive behavior such as sexual, financial, and neglect, deserve further exploring in future research. Last, mental illness stigma is a social process deeply tied to culture, and its influence factors are likely to vary across cultures. The findings of this study conducted in China may not be generalized to other regions.

This study is in no way an attempt to place blame on the family caregivers of PLS or to place more pressure and stigma on their shoulders. Instead, this study calls for more care and support for family caregivers of PLS. Abuse by family caregivers towards PLS should not be simplified as a personal moral issue and roughly identified as family discrimination. Sometimes, it is not discrimination that drives caregivers to commit abusive behaviors against their loved ones. Families may use violence as a means of coping with the PLS’ illness, especially if they lack scientific knowledge about managing schizophrenia. Additionally, if family caregivers are overwhelmed by the caregiving burden and stress, they may treat the PLS roughly as a negative way of coping^17^. This study hopes to highlight the influence of family caregivers’ abusive behavior and bring more attention to this hidden problem. Family caregivers have played a pivotal role in the care of PLS since the advent of deinstitutionalization, and their burden and suffering should be recognized and ameliorated in time. Furthermore, this study aims to inspire future anti-stigma interventions. Nowadays, many interventions have been developed to end the mental illness stigma, most of which were aimed at reducing stigma in the general population or healthcare workers^20^. Few intervention studies consider reducing the stigma of PLS by providing a healthy family environment and high-quality family care. The present study suggested that the application of family interventions for abusive behavior may further reduce the stigma of PLS. To end all forms of stigma and discrimination against PLS, more attention needs to be paid to the families of PLS.

### Supplementary information

Supplementary material

## Data Availability

The data that support the findings of this study are not openly available due to reasons of sensitivity and are available from the corresponding author upon reasonable request.
