# Supplementary material for: Family caregivers’ abusive behaviour and its association with internalized stigma of people living with schizophrenia in China
Source: Schizophrenia (Heidelb). 2023 Sep 19;9(1):61. doi: 10.1038/s41537-023-00393-6 (PMC10509261; doi:10.1038/s41537-023-00393-6)
Supplement: Supplementary file 1 — Supplementary material [file 41537_2023_393_MOESM1_ESM.docx]

Supplementary Table 1. characteristics between the missing data subset and the analyzed population

|  | Included in analysis | | t or chi | p |
| --- | --- | --- | --- | --- |
|  | No | Yes |  |  |
| **Residence** |  |  | 16.698 | 0.001 |
| Urban | 128 (73.1) | 596 (86.0) |  |  |
| Rural | 47 (26.9) | 97 (14.0) |  |  |
| **Gender** |  |  | 2.211 | 0.137 |
| Male | 89 (50.9) | 309 (44.6) |  |  |
| Female | 86 (49.1) | 384 (55.4) |  |  |
| **Age** | 47.22 (13.05) | 45.99 (12.96) | 1.124 | 0.261 |
| **Illness duration** | 18.74 (11.14) | 18.53 (10.70) | 0.230 | 0.818 |
| **Education** |  |  | 1.896 | 0.388 |
| Primary or below | 54 (30.9) | 188 (27.1) |  |  |
| Secondary | 107 (61.1) | 429 (61.9) |  |  |
| College/university | 14 (8.0) | 76 (11.0) |  |  |
| **Marital Status** |  |  | 3.23 | 0.198 |
| Single | 53 (30.3) | 258 (37.2) |  |  |
| Married or cohabiting | 104 (59.4) | 362 (52.2) |  |  |
| Separated divorced Widowed | 18 (10.3) | 73 (10.5) |  |  |
| **BPRS, M(SD)** | 22.30 (8.54) | 22.02 (7.15) | 0.45 | 0.652 |
| **WHO-DAS, M(SD)** | 26.18 (13.98) | 24.00 (12.18) | 2.05 | 0.040 |

Supplementary Table 2. multivariable logistic regression analysis of abusive behavior and level of internalized stigma

|  | OR | 95% confidence intervals | | p |
| --- | --- | --- | --- | --- |
|  |  | Lower | Upper |  |
| **Experienced abusive behavior** |  |  |  |  |
| No | Ref. | Ref. | Ref. |  |
| Yes | 1.77 | 1.20 | 2.63 | 0.004 |
| **Residence** |  |  |  |  |
| Urban | Ref. | Ref. | Ref. |  |
| Rural | 2.17 | 1.31 | 3.60 | 0.003 |
| **Age** | 0.98 | 0.96 | 0.99 | 0.011 |
| **Illness duration** | 1.01 | 0.99 | 1.03 | 0.273 |
| **Gender** |  |  |  |  |
| Male | Ref. | Ref. | Ref. |  |
| Female | 0.89 | 0.63 | 1.26 | 0.508 |
| **Education,** |  |  |  |  |
| Primary or below | Ref. | Ref. | Ref. |  |
| Secondary | 1.16 | 0.77 | 1.74 | 0.481 |
| College/university | 1.16 | 0.61 | 2.20 | 0.658 |
| **Marital Status** |  |  |  |  |
| Separated divorced Widowed | Ref. | Ref. | Ref. | 0.005 |
| Single | 0.40 | 0.22 | 0.75 | 0.004 |
| Married or cohabiting | 0.75 | 0.43 | 1.30 | 0.308 |
| **Employment** |  |  |  |  |
| Unemployed | Ref. | Ref. | Ref. |  |
| Retired | 0.96 | 0.52 | 1.76 | 0.883 |
| Part-time | 2.60 | 1.02 | 6.61 | 0.045 |
| Full-time | 0.72 | 0.44 | 1.20 | 0.214 |
| **Household poverty** |  |  |  |  |
| No | Ref. | Ref. | Ref. |  |
| yes | 0.89 | 0.57 | 1.39 | 0.611 |
| **BPRS** | 1.03 | 1.01 | 1.07 | 0.022 |
| **WHODAS 2.0** | 1.06 | 1.04 | 1.07 | <0.001 |
